# Supplementary material for: Recreational Drugs and the Risk of Hepatocellular Carcinoma
Source: Cancers (Basel). 2022 Nov 2;14(21):5395. doi: 10.3390/cancers14215395 (PMC9657889; doi:10.3390/cancers14215395)
Supplement: Supplementary file 1 [file cancers-14-05395-s001.zip › cancers-1979273-supplementary.pdf]

Supplementary Table S1. Cases of AAS-related liver tumor reported in the literature

Authors	Gender	Age (year-old)	Underlying liver disease	Liver tumor type	Evidence on metastasis	Treatment
Bernstein *et al* [100]	NA	20	No	HCC	No	No
Johnson et *al* [101] (4 cases)	Female	21	No	HCC	No	AAS withdrawal
	Female	17	No	HCC	No	No
	Male	27	Cryptogenic Cirrhosis	HCC	No	No
	Male	21	No	HCC	No	No
Henderson *et al* [102]	Male	4	No	NA	No	No
Farrell GC *et al* [103] (3 cases)	Male	28	No	HCC	No	AAS withdrawal
	Male	40	Alcohol chronic liver disease	HCC	No	Predominant tumor resection and AAS withdrawal
	Male	33	No	HCC	Yes (bone)	AAS withdrawal and palliative radiotherapy (bone metastasis)
Hernández *et al* [104]	Male	19	No	LA	No	Tumor resection
Shapiro *et al* [105]	Male	13	No	HCC	No	No
Lopez *et al* [106]	Female	65	No	HCC	No	NA
Carrasco *et al* [107]	Male	32	No	LA	No	No
Linares *et al* [108]	Female	31	No	HCC	No¥	AAS withdrawal
Bork *et al* [109] (3 cases)	Female	69	No	LA	NA	AAS withdrawal
	Female	29	No	LA	NA	AAS withdrawal
	Male	39	No	LA	NA	AAS withdrawal
Socas *et al* [110] (2 cases)	Male	35	No	LA	No	AAS withdrawal
	Male	23	No	LA	No	AAS withdrawal
Martin *et al* [111]	Male	27	No	LA	No	Hepatic segmentectomy and AAS withdrawal
Hardt *et al* [112]	Male	37	No	HCC	No	AAS withdrawal and tumor resection
Pais-Costa *et al* [113]	Male	28	No	LA	No	Tumor resection and enucleation and AAS withdrawal
Kesler *et al* [114]	Male	24	Hepatic adenomatosis Hemorrhagic adenoma	HCC	No	Transarterial chemoembolization
Solbach *et al* [115]	Male	29	Fatty liver*	HCC	No	Liver transplant
Kato K *et al* [116]	Female	32	Non-alcoholic fatty liver disease	LA	No	Hepatic segmentectomy, enucleation, open surgical radiofrequency (first time) Transarterial chemiembolization and radiofrequency (second time, residual tumors)
Woodward *et al* [117] (2 cases)	Male	39	No	HCC	No	Resection tumor
	Male	28	No	HCC	No	Resection tumor and AAS withdrawal
Wang *et al* [118]	Male	15	No	LA	No	AAS withdrawal
Lin *et al* [119]	Female	17	No	HCC	No¥¥	Local Yttrium-90 therapy (twice) and AAS withdrawal

HCC: hepatocellular carcinoma, LA: liver adenoma, NA: no available. *Fatty liver in abdominal ultrasonography (with no apparent risk factors for non-alcoholic fatty liver disease nor alcohol intake). ¥ Patient suffered from adenopathy metastasis whose primary tumor was a esophagus squamous cell carcinoma (concomitant tumor) ¥¥ Liver magnetic resonance showed portal invasion
